# Suppression of ovine lymphocyte activation by *Teladorsagia circumcincta* larval excretory-secretory products

**DOI:** 10.1186/1297-9716-44-70

**Published:** 2013-08-21

**Authors:** Tom N McNeilly, Mara Rocchi, Yvonne Bartley, Jeremy K Brown, David Frew, Cassandra Longhi, Louise McLean, Jenni McIntyre, Alasdair J Nisbet, Sean Wattegedera, John F Huntley, Jacqueline B Matthews

**Affiliations:** 1 Moredun Research Institute, Pentlands Science Park, Bush Loan, Penicuik EH26 0PZ, UK; 2 MRC Centre for Reproductive Health, Queen’s Medical Research Institute, The University of Edinburgh, 47 Little France Crescent, Edinburgh EH16 4TJ, UK

## Abstract

*Teladorsagia circumcincta* is an important pathogenic nematode of sheep. It has been demonstrated previously that stimulation of murine T lymphocytes with excretory-secretory (ES) products derived from fourth stage larvae of *T*. *circumcincta* (*Tci*-L4-ES) results in de novo expression of Foxp3, a transcription factor intimately involved in regulatory T cell function. In the current study, Foxp3^+^ T cell responses in the abomasum and the effects of *Tci*-L4-ES on ovine peripheral blood mononuclear cells (PBMC) following *T*. *circumcincta* infection were investigated. *T*. *circumcincta* infection resulted in a significant increase in numbers of abomasal Foxp3^+^ T cells, but not an increase in the proportion of T cells expressing Foxp3. Unlike in mice, *Tci*-L4-ES was incapable of inducing T cell Foxp3 expression but instead suppressed mitogen-induced and antigen-specific activation and proliferation of ovine PBMC in vitro. This effect was heat labile, suggesting that it is mediated by protein(s). Suppression was associated with up-regulation of interleukin-10 (IL-10) mRNA, and specific monoclonal antibody neutralisation of IL-10 resulted in a 50% reduction in suppression, indicating involvement of the IL-10 signaling pathway. Suppression was significantly reduced in PBMC isolated from *T*. *circumcincta* infected vs. helminth-naïve lambs, and this reduction in suppression was associated with an increase in *Tci*-L4-ES antigen-specific T cells within the PBMC. In conclusion, we have identified a mechanism by which *T*. *circumcincta* may modulate the host adaptive immune response, potentially assisting survival of the parasite within the host. However, the impact of *Tci*-L4-ES-mediated lymphocyte suppression during *T*. *circumcincta* infection remains to be determined.

## Introduction

*Teladorsagia circumcincta* is a pathogenic nematode of small ruminants and represents a major constraint on farming. The parasite is endemic in temperate regions worldwide and its associated disease, parasitic gastroenteritis, is common. Infections are associated with production losses in lambs, most notably reductions in appetite and live-weight gains, but the parasite can also cause diarrhea, dehydration and death [1]. *T*. *circumcincta* resides in the abomasum, which is analogous to the monogastric stomach, and sheep become infected by ingestion of infective third stage larvae (L3) from pasture. These invade the gastric glands where they develop to fourth stage larvae (L4) and fifth stage larvae (L5) after approximately 10 days. The L5 re-emerge into the lumen to complete development to adult worms around 18 days post-infection (dpi), with egg laying starting at 18–21 dpi. Reductions in appetite and weight gain have been largely attributed to, as yet, undefined components of the anti-parasite immune response rather than as a consequence of damage to host tissue by the parasite *per se*[2]. That said, severe parasite-induced histopathological changes do occur in the abomasum of infected animals [3]. Globally, control of parasitic gastroenteritis relies heavily on anthelmintics; however, drug resistance in *T*. *circumcincta* is widespread and ever-increasing [4] and alternative methods of control are urgently required.

Although protective immunity to *T*. *circumcincta* does occur, it requires continuous infection over a number of weeks to develop [5] and, in practice, is not sufficiently rapid to prevent substantial pasture contamination resulting in major losses in production and clinical disease within the same grazing season. In the absence of further parasite challenge, elements of the protective response which can, for example, result in the induction of inhibited L4, are comparatively short-lived, requiring continuous exposure to *T*. *circumcincta* to be maintained [6]. This relative delay in acquisition of immunity, as well as the somewhat incomplete nature of the protective response, suggests that, as with other nematode species [7,8], *T*. *circumcincta* may actively suppress host immune responses facilitating survival within the host. While the precise effector mechanisms of protective immunity are not fully understood, it is thought to involve both innate and adaptive responses (reviewed in [9]). Local *T*. *circumcincta* specific IgA appears to play a key role, with significant negative correlations reported between local IgA levels and L3 establishment, L4 development and adult length and fecundity [10,11]. Further evidence of a role for local adaptive responses was obtained in experiments whereby lymphoblasts in gastric efferent lymph derived from immune, previously-infected lambs were found to confer protective immunity and memory IgA responses when transferred to helminth-free recipient lambs [12]. Cytokine mRNA profiles in abomasal lymph nodes derived from infected sheep suggest that, in common with other nematode infections [7], the effector response to *T*. *circumcincta* is largely Th2- type in nature, in concert with a regulatory-type response [13].

Parasitic nematodes regulate host immune responses through a number of mechanisms including interference with antigen processing, modulation of macrophage and antigen-presenting cell function, interference with cytokine signaling, or induction of immunoregulatory cell types (reviewed in [14]). In many cases, immunosuppressive activity has been attributed to molecules that are excreted or secreted by the nematodes [8]. It was shown recently that excretory-secretory (ES) products from *T*. *circumicincta* L4 induce de novo expression of Foxp3, a transcription factor intimately involved in regulatory T cell (Treg) function, in activated murine CD4^+^ T lymphocytes in vitro [15], suggesting that the parasite may actively induce regulatory T cell responses during infection. Studies on *Ostertagia ostertagi*, a closely related nematode of cattle, demonstrated that its larval stages can suppress lymphocyte activation: peripheral blood lymphocyte proliferative responses to the mitogen, phytohaemagglutinin, were transiently depressed during the prepatent period of infection [16], whilst soluble somatic L4 extracts and L4 ES products from *O*. *ostertagi* were found capable of suppressing mitogen-induced bovine lymphocyte proliferation in vitro [17]. Whether larval stages of *T*. *circumcincta* are similarly capable of modulating the ovine immune response is currently unknown. The aim of this study was to determine whether Foxp3^+^ T cells increase during *T*. *circumcincta* infection and, secondly, to explore the capacity of larval ES products to modulate ovine lymphocyte responses.

## Materials and methods

### Animals and *T*. *circumcincta* challenge models

Animal procedures were performed at Moredun Research Institute (MRI) under license as required by the UK Animals (Scientific Procedures) Act 1986, and ethical approval was obtained from the MRI Animal Experiments Committee. With the exception of ovalbumin-immunized lambs, all animals were raised at MRI under conditions designed to exclude accidental infection with helminth-parasites, and were considered helminth-naïve. To provide abomasal mucosal tissue for subsequent immunohistochemical (IHC) analyses, twelve yearling Suffolk-cross lambs were infected with 50 000 *T*. *circumcincta* L3 and abomasal mucosa collected at post-mortem at five (*n* = 6) and ten (*n* = 6) days post-infection. Abomasal mucosa was collected from a further six, age and breed-matched, helminth-naïve lambs to serve as uninfected controls. For general provision of peripheral blood mononuclear cells (PBMC), blood was collected from six, 6–12 month-old Scottish Blackface cross lambs via jugular venepuncture at a frequency of no greater than two occasions every four weeks. To determine the effects of parasite ES products on antigen-specific lymphocyte responses, PBMC were purified from three, 9 month-old Bluefaced Leicester × Blackface cross lambs which had been immunized three months previously with 60 μg low-endotoxin ovalbumin (EndoGrade^®^ Ovalbumin, Hyglos GmbH, Bernried am Starnberger See, DE) plus 5 mg Quil A (Brenntag Biosector, Frederikssund, DK) on two separate occasions two weeks apart via the intramuscular route. For isolation of PBMC during the course of a *T*. *circumcincta* infection, seven, 7 month-old Texel-cross lambs were infected with 2000 *T*. *circumcincta* L3 three times a week for four weeks. Faecal samples were collected before challenge and three times a week from day 12 after the first challenge and faecal egg counts (FECs) performed as previously described [18]. Identification to species of parasite eggs within faecal samples was performed using species-specific PCR amplification of the ITS-2 region of ribosomal DNA from hatched first stage larvae [19]. Blood was collected by jugular venepuncture at 0, 2, 4 and 6 weeks from the start of infection for subsequent isolation of PBMC. To determine ES antigen-specific lymphocyte proliferation following infection, PBMC were collected from five 4-month old Texel-cross lambs before and after infection with 2000 *T*. *circumcincta* L3 three times a week for six weeks.

### Production of ES products from *T*. *circumcincta* fourth stage larvae (L4)

Helminth-free Bluefaced Leicester × Blackface lambs (< 6 months-old) were infected with 50 000 *T*. *circumcincta* L3 and mucosal stage L4 harvested at 7 dpi following previously described methods [20]. The parasites were washed three times in PBS before culturing in RPMI 1640 (Invitrogen, Carlsbad, CA, USA) containing 1% (v/v) D-glucose, 2mM L-glutamine, 100 U/mL penicillin, 100 μg/mL streptomycin, 250 μg/mL gentamycin and 125 μg/mL amphotericin B, at 37 °C in 5% CO_2_. Culture supernatants were harvested after 24 h and the media replenished. Parasites were cultured for a further 24 h, when the supernatants were collected and the parasites discarded. Viability of the parasites was confirmed at the end of the culture period on the basis of structural integrity and motility. The culture supernatants were clarified by centrifugation, then passed through 0.2 μm sterile filters. L4 ES products were subsequently concentrated approximately 40-fold using an Amicon Ultra-15 centrifugal filter with a 10 kDa cut-off (Sigma-Aldrich, St. Louis, MO, USA). Protein concentrations were assessed using a Pierce BCA protein assay kit according to the manufacturer’s instructions (Thermo Scientific) and aliquots stored at −80 °C prior to use. For some experiments, endotoxin was removed from the L4 ES products using an EndoTrap^®^ red column according to the manufacturer’s instructions (Hyglos GmbH). Endotoxin levels were quantified using a LAL Chromogenic Endpoint Assay kit (Hycult^®^biotech, Uden, The Netherlands) and were 3 × 10^5^ EU/mg before endotoxin removal and <100 EU/mg after removal.

### Immunohistochemistry

Abomasal mucosal tissue was fixed in 4% paraformaldehyde in PBS and paraffin-embedded. Tissue sections were cut to 5 μm thick and mounted on Superfrost Plus glass slides (Thermo Fisher Scientific, Waltham, MA, USA). Single IHC labeling of Foxp3 and CD3 was performed as follows: sections were rehydrated and antigen-retrieval performed by autoclaving at 121 °C for 10 min in 10 mM citrate buffer, pH 6.0. Slides were incubated in 0.3% hydrogen peroxide for 20 min at RT to quench endogenous peroxidase activity followed by blocking in 25% normal goat serum (NGS) in PBS/0.5% Tween 80 (PBS/T80) for 1 h at RT. Sections were then incubated overnight at 4 °C with 5 μg/mL of mouse anti-human Foxp3 monoclonal antibody (mAb) 22510 (mAbcam 22510, Abcam, Cambridge, UK) which had previously been shown to cross-react with ovine Foxp3 [21], 2 μg/mL polyclonal rabbit anti-human CD3 antibody (Dako UK Ltd., Ely, UK) or the appropriate controls (mouse anti-Border Disease virus p125/p80 IgG1 mAb and purified rabbit immunoglobulin (Sigma-Aldrich) for mouse and rabbit primary antibodies, respectively). All antibodies were diluted in PBS/T80 containing 10% NGS. After washing, sections were incubated with the appropriate secondary antibody (peroxidase-labelled anti-mouse or anti-rabbit EnVision™ + reagent; Dako) for 30 min at RT before developing with 3,3′-diaminobenzidine, counterstaining with haematoxylin, dehydrating and mounting in Shandon synthetic mountant (Thermo Fisher Scientific). For each run of IHC, a section of gastric lymph node was analyzed in parallel as a positive control. For dual fluorescence labeling of Foxp3 and CD3, following antigen retrieval and blocking, sections were incubated overnight at 4 °C with both 5 μg/mL mAbcam 22510 (Abcam) and 2 μg/mL polyclonal rabbit anti-human CD3 antibody or the appropriate controls (VPM21 and purified rabbit immunoglobulin). Sections were then incubated with horseradish peroxidase (HRP) labeled anti-mouse EnVision™ + reagent (Dako) for 30 min at RT and Foxp3 signal developed by incubation with Alexa Fluor^®^ 488 tyramide substrate (Invitrogen). Following washing, CD3 signal was developed by incubation with 2 μg/mL donkey anti-rabbit IgG (H+L) conjugated to Rhodamine Red-X (Jackson ImmunoResearch Laboratories Inc., West Grove, PA, USA) and slides mounted in Mowiol 4–88 (Calbiochem, Nottingham, UK). Cell counts were performed on single-labeled sections by counting the number of positive cells within the abomasal mucosa in 10 non-overlapping fields at ×20 magnifications using a Leitz Dialux 20 light microscope (Leica Microsystems (UK) Ltd., Milton Keynes, UK) fitted with a 450 × 450 μm graticule. Counts were expressed as numbers of cells per mm^2^ of abomasal mucosa. To determine the proportion of CD3^+^ cells which were also Foxp3^+^, dual fluorescent labeled slides were analyzed using an Axiovert 200M inverted fluorescence microscope equipped with an ApoTome slider module (Carl Zeiss Ltd., Welwyn Garden City, UK). A total of 500 CD3^+^ cells were analyzed per section and the number of cells which were double positive for CD3 and Foxp3 recorded. For publication purposes, images were captured using a Zeiss 710LSM confocal microscope (Carl Zeiss Ltd).

### Lymphocyte stimulation assays

Blood was collected into EDTA-containing Vacutainers (BD, Franklin Lakes, NJ, USA) and PBMC isolated using a Ficoll-Paque™ PLUS density gradient (GE Healthcare, Little Chalfont, UK) according to the manufacturer’s instructions. PBMC were cultured in 96-well round-bottomed plates at 5 × 10^5^ cells/well in RPMI 1640 (Invitrogen) containing 10% foetal calf serum (FCS), 2 mM L-glutamine, 100 U/mL penicillin, 100 μg/mL streptomycin and 50 μM 2-mercaptoethanol at 37 °C 5% CO_2_. To determine the effects of L4 ES products on mitogen-induced proliferation, *T*. *circumcincta* L4 ES products (*Tci*-L4-ES) or an equivalent volume of PBS were added for 30 min followed by addition of 5 μg/mL Concanavalin A (Con A, Sigma-Aldrich). *Tci*-L4-ES that had been previously heat-treated for 30 min at 100 °C was used as a control in initial experiments. These heat-treatment conditions have been previously been reported for nematode ES products [15] and were used to ensured complete denaturation of protein components within the ES. After 72 h, cells were harvested for flow cytometric analysis or for preparation of RNA for subsequent quantitative PCR analysis. In some experiments, naïve CD4^+^CD25^-^CD45RA^+^ T cells plus irradiated autologous antigen-presenting cells were used instead of PBMC. For time course studies, cells were also harvested at 0, 24, 48 and 72 h post-stimulation for flow cytometry analysis. Cell proliferation was measured by incorporation of methyl-3H-thymidine ([^3^H] thymidine, 0.5 μCi per well; GE Healthcare) for the final 18 h of culture, after which cells were harvested onto glassfibre filters (Perkin-Elmer, Cambridge, UK) and counted in a Wallac 1450 MicroBeta^®^ TriLux Microplate Scintillation and Luminescence Counter (Perkin-Elmer). Proliferation results were expressed as counts per minute (cpm). In some experiments, cell-free culture supernatants were harvested at 54 h of culture and stored at −70 °C for subsequent cytokine ELISAs.

To exclude possible apoptotic and/or necrotic effects, cells were also assessed in some cultures at 72 h using both an Annexin V APC apoptosis detection kit (BD Pharmingen™, San Diego, CA, USA), in which Annexin V+/7AAD- labeling indicates early apoptosis and Annexin V+/7AAD+ labeling indicates either late apoptosis or necrosis, and a Caspase 3 activity assay kit (Roche Applied Science, Burgess Hill, UK), according to the manufacturers’ instructions.

To determine the role of interleukin-10 (IL-10) PBMC were cultured in the presence of 5 μg/mL anti-ovine IL-10 monoclonal antibody (mAb) CC320 (AbDserotec, Kidlington, UK) or 5 μg/mL mouse IgG1 isotype control (eBioscience). To determine the effects of *Tci*-L4-ES on antigen-specific lymphocyte proliferation, PBMC were cultured as described above with 10 μg/mL low-endotoxin ovalbumin (EndoGrade^®^ Ovalbumin, Hyglos GmbH) in the presence or absence of 30 μg/mL *Tci*-L4-ES. Proliferation was assessed after 5 days of culture by the measurement of methyl-3H-thymidine incorporation. To determine proliferation and IL-10 release in response to ES antigens, PBMC were incubated with 30 μg/mL heat-inactivated *Tci*-L4-ES and culture supernatants were harvested at 54 h and proliferation assessed at 72 h. Proliferation results were expressed as a Stimulation index (SI) which was calculated as the cpm of *Tci*-L4-HiES stimulated PBMC divided by the cpm of unstimulated control PBMC.

### Flow cytometry and fluorescence-activated cell sorting (FACS)

Triple labeling of PBMC for CD4, CD25 and Foxp3 was performed using the following monoclonal antibodies (mAb): 1 μg/mL anti-ovine CD4 conjugated to FITC (clone 44.38, mouse IgG2a, AbDserotec), 2 μg/mL anti-ovine CD25 conjugated to R-phycoerythrin (RPE) (clone ILA111, mouse IgG1, AbDserotec), 2.5 μg/mL anti-mouse/rat Foxp3 conjugated to Alexa Fluor^®^ 647 or RPE-Cy7 (clone FJK-16s, rat IgG2a, eBioscience), or the appropriate isotype controls, as previously described [22]. Briefly, cells were incubated with anti-ovine CD4-FITC and anti-ovine CD25-RPE in FACS buffer (PBS + 5% FCS) for 30 min at 4 °C, then fixed in 1% paraformaldehyde in PBS for 10 min at room temperature (RT). Following fixation, cells were permeabilised overnight at 4 °C in FACS buffer containing 0.2% saponin (Sigma-Aldrich) and 20% normal rat serum. Cells were then incubated with anti-mouse/rat Foxp3 antibody in FACS buffer for 1 h at 4 °C and fixed again in 1% paraformaldehyde in PBS for 10 min prior to analysis. For each experiment, previously generated Chinese hamster ovary cells transfected with ovine Foxp3 were labeled in parallel as a positive control for Foxp3 staining. Cells were analysed on a Cyan™ ADP flow cytometer (Beckman Coulter Inc., Fullerton, CA, USA) using the manufacturer’s acquisition software (Summit version 4.3). Cells were gated on the basis of their physical characteristics (forward and side scatter) to exclude debris and damaged cells. For FACS sorting of naïve CD4^+^ T cells, PBMC were labeled with anti-ovine CD45RA (clone 73B, mouse IgG1 [23]) followed by anti-mouse IgG1 conjugated to Alexa Fluor^®^ 647 (Invitrogen) and then blocked in 20% normal mouse serum before incubating with anti-ovine CD4-FITC and anti-ovine CD25-RPE. Naïve CD4^+^CD25^-^CD45RA^+^ cells were sorted using a BD FACSAria™ IIIu cell sorter (BD Biosciences, San Jose, CA, USA), obtaining ~88% purity. CD4^-^ CD45RA^+^ cells were also collected for use as a source of antigen presenting cells in lymphocyte stimulation assays. Analysis of flow cytometry data was performed using FlowJo version 7.6.1 analysis software (TreeStar, San Carlos, CA, USA).

### Quantative RT-PCR analysis

RNA was prepared from cells using an RNeasy Plus kit (Qiagen) which included removal of genomic DNA using a gDNA eliminator column (Qiagen). Relative quantification of gene transcription by two-step, quantitative RT-PCR was performed for IL-4, IL-10, interferon-gamma (IFN-γ) and TGF-β1 using the standard curve method and GAPDH for normalization purposes with modifications [24,25]. Briefly, first strand cDNA was synthesized using Superscript™ II (Invitrogen) and oligo(dT) primers (Sigma-Aldrich) according to the manufacturer’s instructions. Real-time RT-PCR was performed using an ABI Prism 7000 real-time thermal cycler (Applied Biosystems, Foster City, CA, USA) and the appropriate primer sets (Table 1). Cycling was performed in 25 μL reaction volumes using 1 μL cDNA, 12·5 μL of SYBR^®^ GREEN ER™ qPCR Supermix (Invitrogen) and 0·2 μM of each primer. A no-template (negative) control was also included in each assay. Thermal cycling parameters involved a 10 min pre-incubation step at 95 °C followed by 40 cycles of 95 °C for 30 s, 55 °C for 30 s and 72 °C for 30 s. Fluorescence data acquisition was performed after each cycle and melting curve analysis performed at the end of each run to verify the specificity of each PCR product. Serial, 10-fold dilutions (from 10^2^ to 10^8^ copies per μL) of previously constructed plasmids containing the relevant gene (IL-4 [26], IFN-γ [27], IL-10 and TGF-β1 [25]) were amplified in parallel with each series of samples, allowing the automatic generation of standard curves using the Applied Biosystems 7000 System SDS software. The number of copies per μL of cDNA was then calculated and the results normalized to GAPDH.

**Table 1:** Primer sequences and annealing temperatures for real-time PCR assays.

**Gene**	**Genbank accession no.**	**Primer sequence** (**5**′-**3**′)	***T***_**ANN**_ (°**C**)
GAPDH [25]	AF030943	F: GGT GAT GCT GGT GCT GAG TA	57
		R: TCA TAA GTC CCT CCA CGA TG	
IL-4 [28]	AF172168	F: AGA GAT CAT CAA AAC GCT GAA	55
		R: GTC TGC TAC AGG CAG CTC	
IL-10 [25]	NM_001009327	F: TGA AGG ACC AAC TGA ACA GC	55
		R: TTC ACG TGC TCC TTG ATG TC	
TGF-β1 [25]	NM_001009400	F: GAA CTG CTG TGT TCG TCA GC	55
		R: GGT TGT GCT GGT TGT ACA GG	
IFN-γ [27]	NM_001009803	F: CTA AGG GTG GGC CTC TTT TC	55
		R:CAT CCA CCG GAA TTT GAA TC	

*T*_ANN_ = primer annealing temperature.

### Quantification of *T*. *circumcincta* ES-specific antibodies by ELISA

*Tci*-L4-ES-specific IgA and IgG were quantified in serum by indirect ELISA as follows: Microtitre plates were coated overnight at 4 °C with 5 μg/mL *Tci*-L4-ES in 0.1 M carbonate buffer pH 9.6. After washing in PBS, pH 7.4, containing 0.05% Tween 20^®^ (PBS/T), non-specific binding sites were blocked with Tris-buffered saline (TBS) containing 0.05% Tween 20^®^ (TBST) and 5% soya milk powder. Plates were subsequently incubated for 1 h at RT with serum diluted 1:10 for IgA and 1:1000 for IgG in TBST. For each plate, 8 wells were incubated with buffer alone and a known positive control sample was analyzed. After washing, plates were incubated for 1 h at RT with a 1:250 dilution of mouse anti-ovine/bovine IgA (clone K84 2F9, AbDSerotec) or at 1:1000 dilution of anti-sheep/goat IgG-HRP (clone GT-34, Sigma Aldrich) for IgA and IgG ELISAs, respectively. For IgA ELISAs, plates were subsequently incubated with a 1:1000 dilution of rabbit anti-mouse IgG-HRP (Dako). After a final wash in PBS/T, colour reactions were developed by addition of Sigma-Fast OPD substrate (Sigma-Aldrich). Reactions were terminated after 5–10 min by addition of 2.5 M H_2_SO_4_, and the OD at 492nm measured using a Sunrise™ microplate reader (Tecan, Männedorf, CH, Switzerland).

### Quantification of interferon-gamma (IFN-γ), interleukin-4 (IL-4) and IL-10 by ELISA

IFN-γ was quantified using a commercial ELISA kit according to the manufacturer’s instructions (MABTECH AB, Augustendalsvägen, SE, Sweden) and expressed as pg/mL. IL-4 and IL-10 were quantified using paired antibodies (AbD Serotec) and established protocols as previously described [29,30]. Quantification was performed using recombinant ovine IL-10 or ovine IL-4 expressed in Chinese Hamster Ovary (CHO) cells using the pEE14^®^ expression vector (Lonza^™^) according to a previously published protocol [31]. ELISA values were related back to the known biological activity of the cytokines to enable cytokine quantification. IL-4 quantities were expressed as pg/mL and IL-10 quantities expressed as biological units (bU)/mL.

### Statistical analyses

Data was analysed using GraphPad Prism version 5.01. Data were log transformed as required to ensure that observations within each group had an approximately normal distribution with a common variance. Subsequent analysis was performed using one-way ANOVA followed by the Tukey post hoc test for pairwise comparison of means with the exception of data obtained from repeated PBMC cultures from the same animals, which were analysed using repeated-measures two-way ANOVA followed by the Bonferroni post hoc test for comparison of means where data was obtained at multiple time points, or a paired students *t*-test where only two time points were analysed. *P* values of < 0.05 were considered significant.

## Results

### Primary infection with *T*. *circumcincta* results in recruitment of Foxp3^+^ T cells into the abomasal mucosa

**Figure 1:**
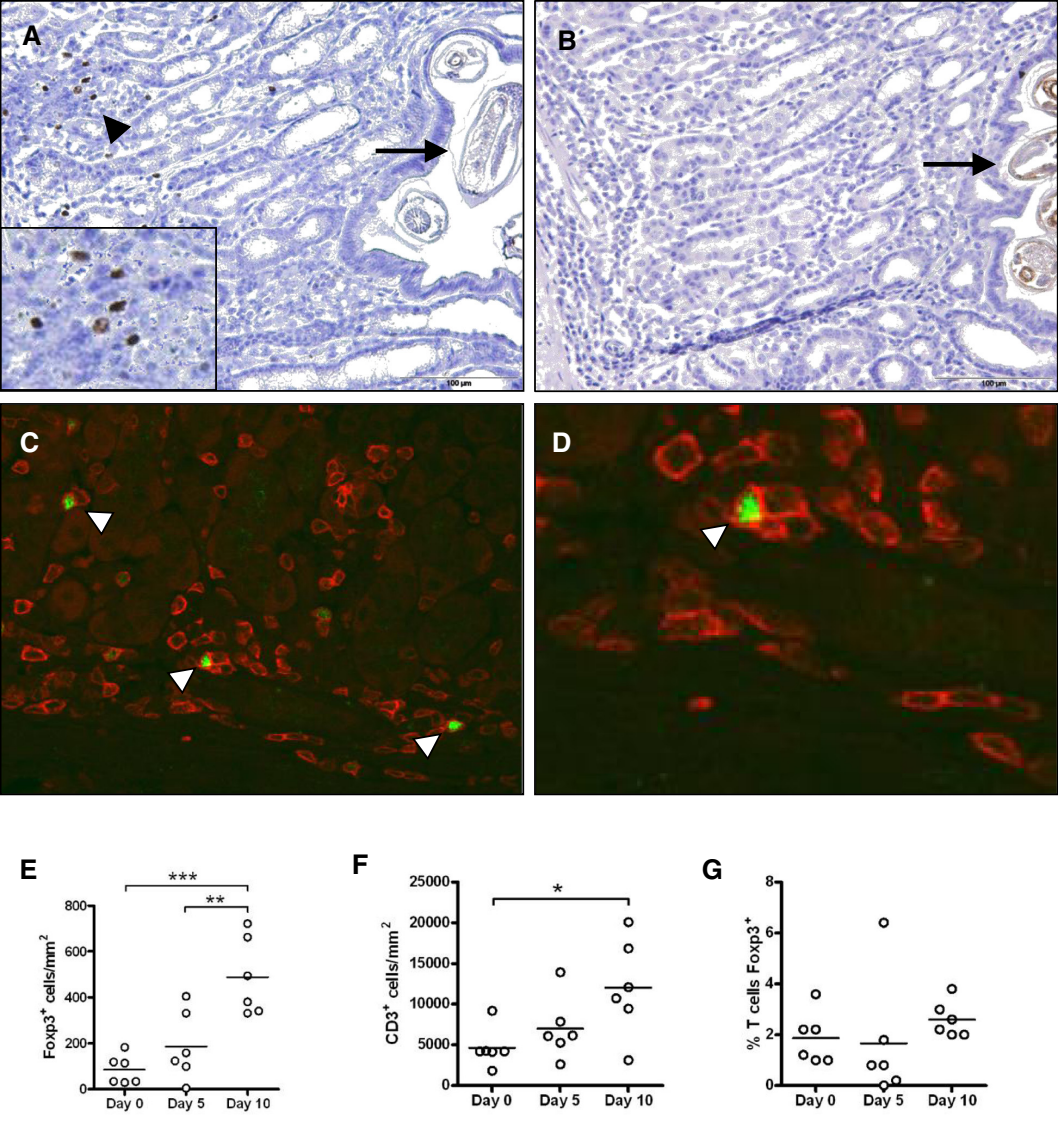
**Immunohistochemical identification of Foxp3**^+ ^**T cells within the abomasal mucosa during infection with *T***. ***circumcincta. *(A)** Single immunohistochemical (IHC) labeling of Foxp3 demonstrating a number of positive cells within the abomasal mucosa at 10 dpi (arrowhead). A high-power view of Foxp3^+^ cells is inset in the panel. The arrow indicates a *T*. *circumcincta* larva within the mucosa. **(B)** Isotype control. **(C)** Double IHC labeling of Foxp3 (green) and CD3 (red) in the abomasal mucosa at 10 dpi. Arrowheads indicate double positive cells. **(D)** High-power view of panel C demonstrating surface labeling of CD3 and nuclear labeling of Foxp3. **(E)** Numbers of Foxp3^+^ cells within the abomasal mucosa at 0, 5 and 10 dpi. **(F)** Numbers of CD3^+^ cells within the abomasal mucosa at 0, 5 and 10 dpi. **(G)** The proportions of CD3^+^ T cells within the abomasal mucosa expressing Foxp3 at 0, 5 and 10 dpi. *P < 0.05, **P < 0.01, ***P < 0.001 (one way ANOVA followed by the Tukey *post hoc* test for pairwise comparison of means). Panel **(A)** has been previously presented by the authors [9].

To determine whether Foxp3^+^ T cell levels increase locally during early infection, helminth-free lambs were challenged with a single infection of 50 000 *T*. *circumcincta* L3 and the numbers and location of Foxp3^+^ T cells within the abomasal mucosa determined at 0, 5 and 10 days post-challenge by IHC. Representative IHC labeling is shown in Figure 1A-D, and cell count data shown in Figure 1E-F. A significant increase in numbers of abomasal mucosal Foxp3^+^ cells was observed at 10 dpi (*P* < 0.001, Figure 1E). Positive cells were often present in clusters adjacent to, but not in direct contact with, mucosal stage larvae (Figure 1A). The increase in Foxp3^+^ cells mirrored the increase in abomasal CD3^+^ T cell numbers, which were also significantly increased at 10 dpi (*P* < 0.05, Figure 1F). The proportion of abomasal T cells expressing Foxp3, as determined by double IHC labeling of CD3 and Foxp3, did not change significantly over the 10-day period (Figure 1G). Furthermore, all Foxp3^+^ cells within the abomasal mucosa were also CD3^+^, confirming their identity as T cells.

### *T*. *circumcincta* L4 ES products inhibit ovine lymphocyte activation in vitro

**Figure 2:**
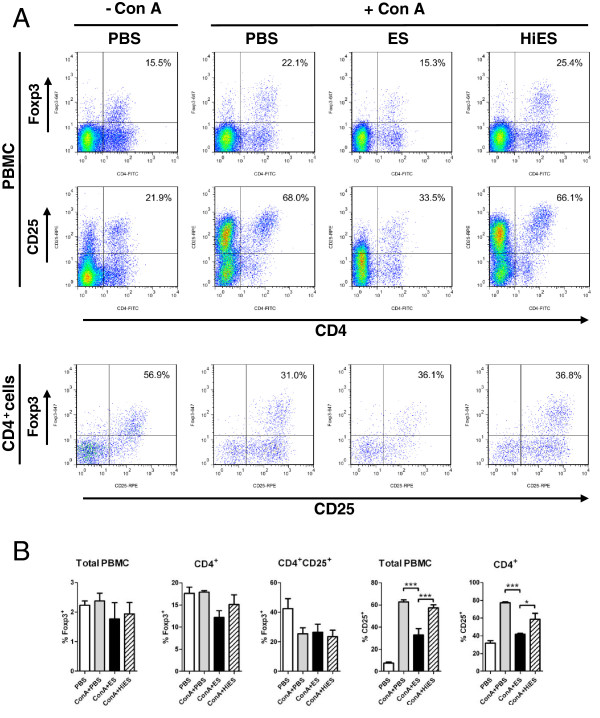
**Expression of Foxp3 and CD25 by CD4**^**+ **^**T cells stimulated with Con A ± *Tci*-L4-ES. (A)** Representative plots of Foxp3 and CD25 expression by CD4^+^ T cells from PBMC from helminth-naïve lambs cultured for 72 h with PBS alone (PBS), 5 μg/mL Con A alone (ConA + PBS), 5 μg/mL Con A + 30 μg/mL *Tci*-L4-ES (ConA + ES) or 5 μg/mL Con A + 30 μg/mL heat-inactivated *Tci*-L4-ES (ConA + HiES). Numbers indicate the percentage of CD4^+^ cells co-expressing Foxp3 (top panel) or CD25 (middle panel) and the percentage of CD4^+^CD25^+^ cells co-expressing Foxp3 (lower panel). **(B)** Percentages of total PBMC, CD4^+^ cells and CD4^+^CD25^+^ cells co-expressing Foxp3, and percentages of total PBMC and CD4^+^ cells co-expressing CD25. Data represents mean ± SEM from three replicate cultures from three helminth-free lambs. *P < 0.05, ***P < 0.001 (one way ANOVA followed by the Tukey *post hoc* test for pairwise comparison of means).

Having shown that abomasal Foxp3^+^ T cells numbers increase by 10 dpi, a time at which most *T*. *circumcincta* would be at L4 stage in the gastric gland [32], we determined whether *Tci*-L4-ES products were capable of inducing expression of Foxp3 in activated ovine CD4^+^ lymphocytes, as has been previously shown in murine CD4^+^ cells [15]. PBMC cultures from helminth-naïve lambs were stimulated with the T cell mitogen Con A in the presence or absence of 30 μg/mL *Tci*-L4-ES for 72 h, an identical ES concentration and time-point to that used in the murine study [15], and the number of cells expressing Foxp3 and the T cell activation marker CD25 (IL-2R α-chain) determined by flow cytometry. As Foxp3 is preferentially expressed by CD4^+^CD25^+^ T cells [22], Foxp3 expression was determined in both CD4^+^ and CD4^+^CD25^+^ subpopulations. Results are shown in Figure 2. No increase in the proportion of cells expressing Foxp3 within the total PBMC, CD4^+^ or CD4^+^CD25^+^ populations was observed following incubation with *Tci*-L4-ES; however, incubation with *Tci*-L4-ES resulted in a significant decrease in the proportion of cells expressing CD25 in both CD4^+^ and total PBMC populations compared to cultures incubated with Con A alone. This effect was heat labile, as heat-inactivated *Tci*-L4-ES had no effect on the proportion of cells expressing CD25 in Con A-stimulated cultures. These results suggested that *Tci*-L4-ES was inhibiting mitogen-induced T cell activation.

**Figure 3:**
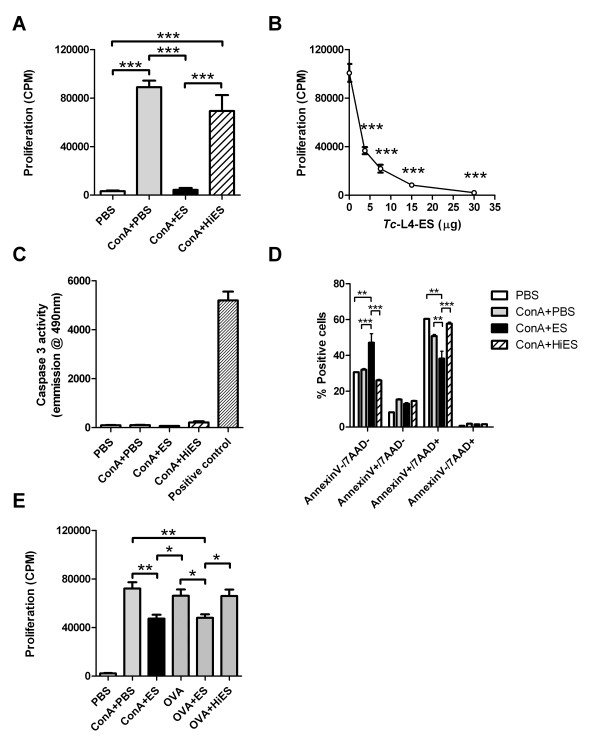
**Suppression of mitogen-induced and antigen-specific lymphocyte proliferation by *Tci*-L4-ES.** To determine the effects of *Tci*-L4-ES on mitogen-induced proliferation, PBMC from helminth-naïve lambs were cultured with Con A in the presence or absence of *Tci*-L4-ES (ES) or heat-inactivated *Tci*-L4-ES (HiES). To determine the effects of *Tci*-L4-ES on antigen-specific proliferation, PBMC from ovalbumin (OVA)-immunized lambs were cultured with OVA in the presence or absence of ES or HiES. Proliferation assessed by incorporation of [3H] thymidine and expressed as counts per minute (cpm). **(A)** Proliferation of helminth-naïve PBMC at 72 h following culture with PBS alone (PBS), 5 μg/mL Con A (ConA + PBS), 5 μg/mL Con A + 30 μg/mL ES (ConA + ES) or 5 μg/mL Con A + 30 μg/mL HiES (ConA + HiES). **(B)** PBMC proliferation at 72 h following culture with 5 μg/mL Con A + 0, 3.75, 7.5, 15 and 30 μg/mL ES. **(C**-**D)** Caspase activity in PBMC cell lysates and Annexin V/7AAD staining in PBMC following 72 h culture with PBS, Con A, Con A + ES or Con A + HiES. **(E)** Proliferation of PBMC from ovalbumin-immunized lambs at 120 h following culture with PBS alone (PBS), 5 μg/mL Con A (ConA + PBS), 5 μg/mL Con A + 30 μg/mL ES (ConA + ES), 10 μg/mL OVA (OVA), 10 μg/mL OVA + 30 μg/mL ES (OVA + ES) or 10 μg/mL OVA + 30 μg/mL HiES (OVA + HiES). Data represents mean ± SEM from three replicate cultures from three helminth-free lambs. *P < 0.05, **P < 0.01, ***P < 0.001 (one way ANOVA followed by the Tukey *post hoc* test for pairwise comparison of means). In panel **(B)** only significant differences between ES-treated and non-ES treated cultures are indicated.

To investigate the potential effects of *Tci*-L4-ES on lymphocyte activation further, we determined the level of cell proliferation in Con A and Con A + ES stimulated cultures at 72 h by incorporation of [^3^H] thymidine. Addition of 30 μg/mL *Tci*-L4-ES to Con A-stimulated cultures resulted in a highly significant (between 85-90%) reduction in cell proliferation compared to cultures stimulated with Con A alone (*P* < 0.001, Figure 3A). This suppressive effect occurred over a range of *Tci*-L4-ES concentrations, and still occurred at 3.75 μg/mL *Tci*-L4-ES (Figure 3B). Again, the effect was heat-labile (Figure 3A). Removal of LPS from *Tci*-L4-ES had no significant effect on the degree of suppression (see Additional file 1). No significant differences in caspase 3 activity were detected between ConA + ES stimulated cultures compared to all other cultures (Figure 3C), whereas the proportions of annexin V^+^ /7AAD^+^ cells was significantly lower and annexin V^-^ /7AAD^-^ cells significantly higher in ConA + ES stimulated cultures compared to all other cultures (Figure 3D), indicating that suppression of Con A-induced proliferation by *Tci*-L4-ES was not associated with increased apoptosis or necrosis.

To determine whether *Tci*-L4-ES was similarly capable of suppressing antigen-specific lymphocyte proliferation, PBMC obtained from three sheep previously immunized with ovalbumin were cultured with ovalbumin in the presence or absence of 30 μg/mL *Tci*-L4-ES and cell proliferation determined after 5 days of culture. Con A and Con A + ES stimulated cultures were also included as controls. Addition of *Tci*-L4-ES resulted in a significant (between 12-46%) reduction in antigen-specific proliferation (*P* < 0.05, Figure 3E), and the relative degree of ES-mediated suppression was similar in both antigen and Con A-stimulated cultures. The suppressive effects were again heat-labile. The level of suppression of Con A stimulated cultures (31-40% reduction in proliferation) was far less than that observed in the previous experiments where we used helminth-free lambs: it was subsequently identified that all ovalbumin-immunized lambs harbored patent *T*. *circumcincta* infections as determined by species-specific PCR using DNA derived from eggs harvested from their faeces.

### Lack of induction of Foxp3 expression in ovine CD4^+^ T cells by *T*. *circumcincta* L4 ES in vitro

**Figure 4:**
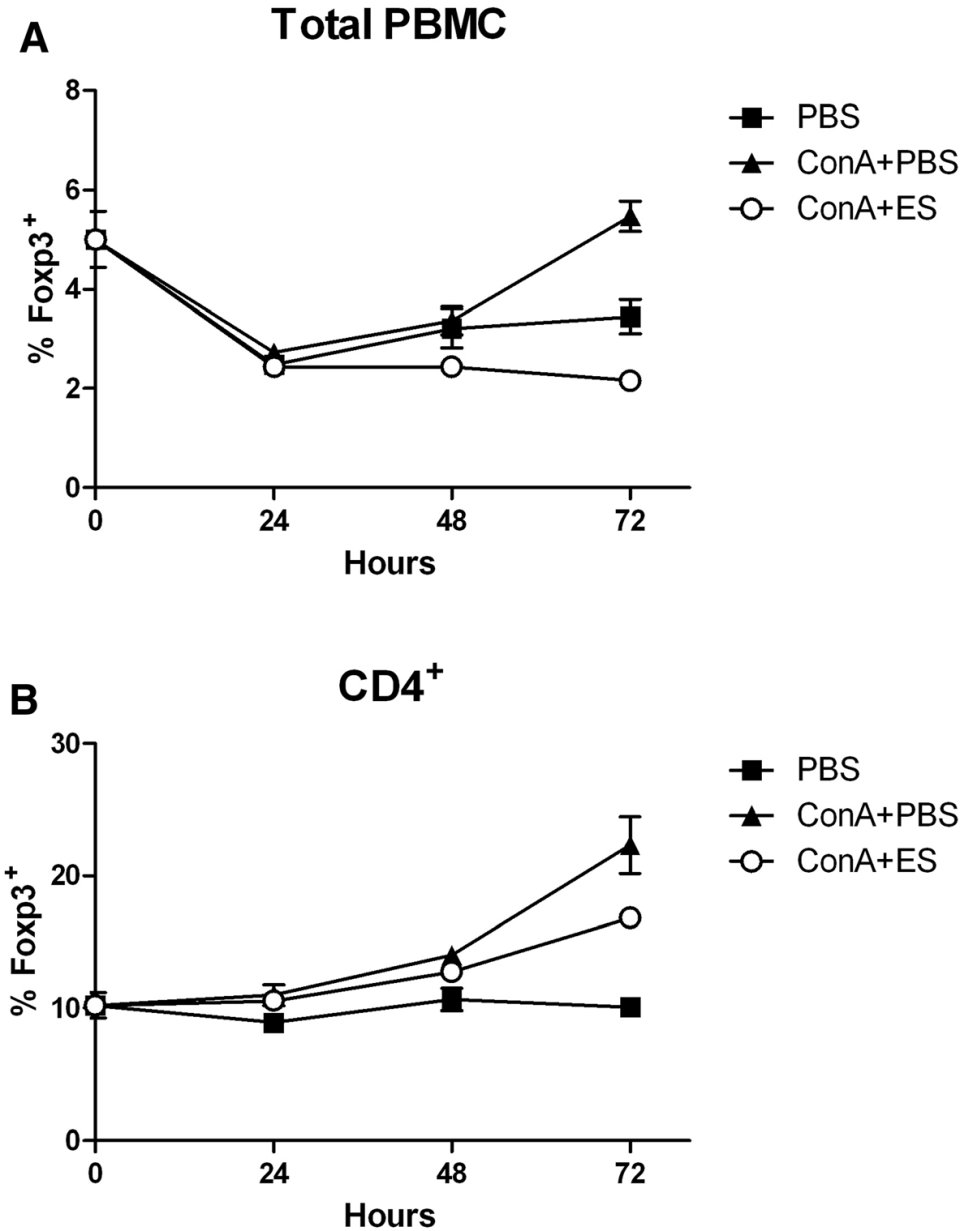
**Time-course of Foxp3 expression in helminth-free PBMC cultures following stimulation with Con A ± *Tci*-L4-ES.** PBMC from a helminth-naïve lamb were cultured with PBS alone (PBS), 5 μg/mL Con A alone (ConA + PBS) or 5 μg/mL Con A + 30 μg/mL *Tci*-L4-ES (ConA + ES). The proportion of Foxp3 positive cells in total PBMC **(A)** and CD4^+^ T cell populations **(B)** was determined at 0, 24 h, 48 h and 72 h by flow cytometry. Data represents mean ± SEM from three replicate cultures.

The lack of observable increase in% Foxp3 expression in the CD4^+^ population in the previous experiments was in contrast to a previous study in mice which demonstrated that activation of CD4^+^ T cells in the presence of 30 μg/mL *Tci*-L4-ES resulted in an ~20% increase in the proportion of CD4^+^ T cells co-expressing Foxp3 by 72 h [15]. One possible reason for this discrepancy is that the dynamics of Foxp3 induction may be different between mice and sheep. We therefore performed a time-course experiment in which PBMC cultures were again stimulated with Con A and *Tci*-L4-ES and expression of Foxp3 determined at 0 h, 24 h, 48 h and 72 h by flow cytometry. The results are shown in Figure 4. No observable increase in the proportion of cells expressing Foxp3 was seen in Con A + ES stimulated cultures, either in the total PBMC population or the CD4^+^ population, compared to cultures incubated with Con A alone at any time-point (Figure 4A-B). As the induction of Foxp3 by *Tci*-L4-ES is thought to be due to the presence of parasite homologues of transforming growth factor-β (TGF-β1), a cytokine which preferentially induces Foxp3 expression in naïve rather than non-naive CD4^+^ T cells [15,33], we also determined whether *Tc*-L4-ES was able to induce Foxp3 expression in purified naïve ovine CD4^+^ T cells. Naïve CD4^+^CD25^-^CD45RA^+^ T cells were stimulated with 30 μg/mL *Tci*-L4-ES in the presence of Con A and irradiated antigen-presenting cells and Foxp3 expression determined at 72 h by flow cytometry. Again, no increase in the proportion of cells expressing Foxp3 was observed in cultures stimulated with Con A + ES compared to Con A stimulation alone (see Additional file 2).

### Suppression of ovine lymphocyte activation by L4 ES products is associated with up-regulation of IL-10 gene expression and involves the IL-10 signaling pathway

**Figure 5:**
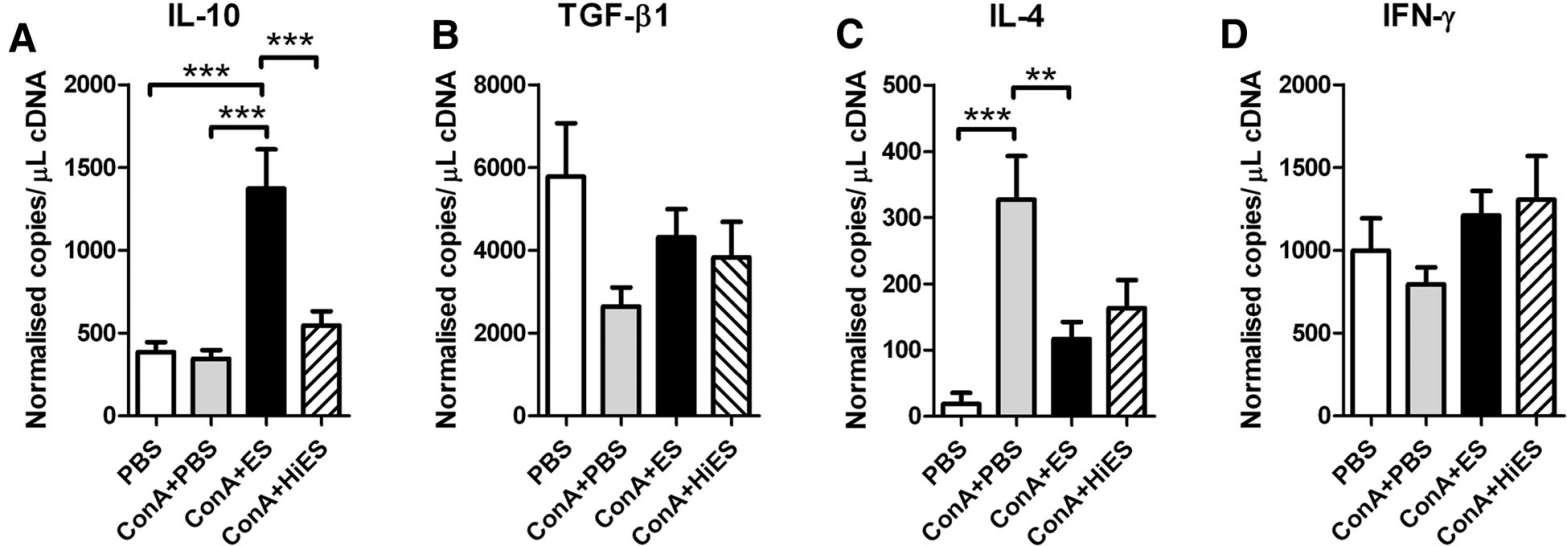
**Gene expression in helminth-free PBMC cultures following stimulation with Con A ± *Tci*-L4-ES.** PBMC from helminth-naïve lambs cultured for 72 h with PBS alone (PBS), 5 μg/mL Con A alone (ConA + PBS), 5 μg/mL Con A + 30 μg/mL *Tci*-L4-ES (ConA + ES) or 5 μg/mL Con A + 30 μg/mL heat-inactivated *Tci*-L4-ES (ConA + HiES). Relative gene expression of *IL*-*10 ***(A)**, *TGF*-β*1 ***(B)**, *IL*-*4 ***(C)** and *IFN*-γ **(D) **was determined by quantitative RT-PCR. Data represents mean ± SEM from three replicate cultures from three helminth-free lambs. **P < 0.01, ***P < 0.001 (one way ANOVA followed by the Tukey *post hoc* test for pairwise comparison of means).

Having identified profound immunosuppressive effects of *Tci*-L4-ES on ovine lymphocyte activation and proliferation, we next determined relative levels of transcription of regulatory (IL-10, transforming growth factor-β1 (TGF-β1)), Th-1 associated (IFN-γ) and Th-2 associated (IL-4) cytokines in Con A and Con A +ES stimulated PBMC after 72 h of culture by qRT-PCR. The results are shown in Figure 5. Stimulation of PBMC cultures with Con A + ES resulted in a significant increase in levels of IL-10 relative transcription compared to un-stimulated, Con A only-stimulated or Con A + heat-inactivated (Hi) ES-stimulated PBMC (*P* < 0.0001, Figure 5A). Levels of IL-4 transcript were significantly lower in Con A + ES-stimulated PBMC compared to Con A-only stimulated PBMC (*P* < 0.01), but were not significantly different to that observed in Con A + Hi ES-stimulated PBMC (Figure 5C). No significant differences in transcript levels of TGF-β1 or IFN-γ were observed. These results indicate that *Tci*-L4-ES-mediated suppression of Con A–induced lymphocyte activation was associated with an increase in PBMC IL-10 transcription.

**Figure 6:**
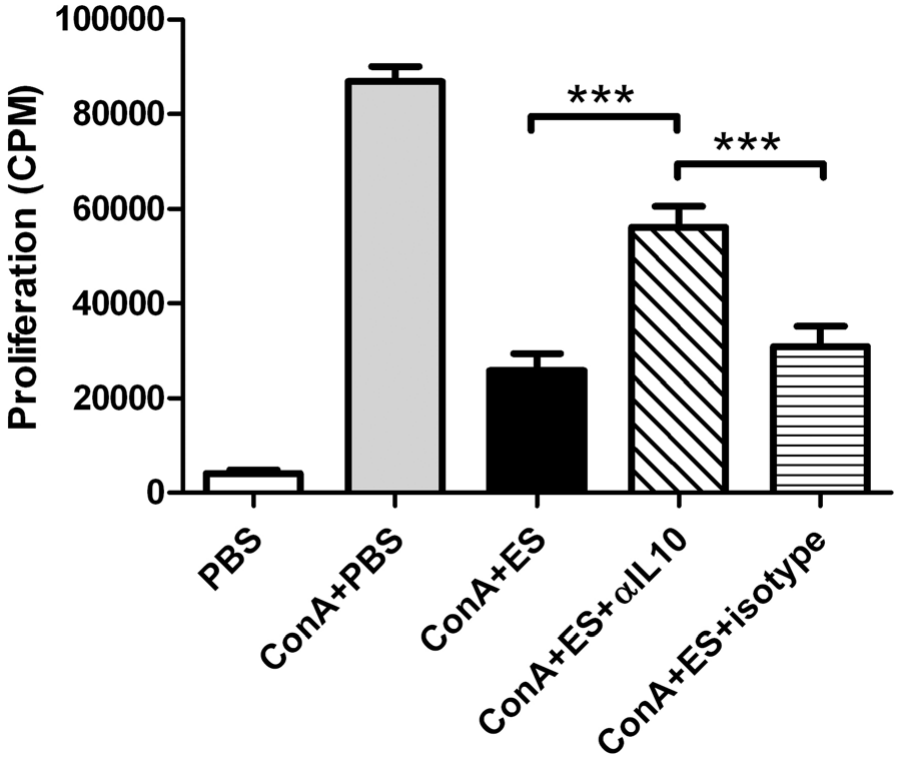
**The effect of IL-10 neutralization on *Tci*-L4-ES mediated lymphocyte suppression.** PBMC from helminth-naïve lambs were cultured with ConA + 15 μg/mL *Tci*-L4-ES in the presence or absence of 5 μg/mL IL-10 neutralizing mAb CC320 (ConA+ES+αIL10) or 5 μg/mL IgG1 isotype control mAb (ConA+ES+isotype). Proliferation was assessed after 72 h by measurement of [3H] thymidine incorporation. Data represents mean ± SEM from three replicate cultures from three helminth-free lambs. ***P < 0.001 (one way ANOVA followed by the Tukey *post hoc* test for pairwise comparison of means).

To determine whether IL-10 signaling was involved in the observed effects, the impact of addition of the IL-10 neutralizing mAb, CC320 [34,35], on *Tci*-L4-ES-mediated suppression of Con A-induced proliferation was determined. The results are shown in Figure 6. Addition of IL-10 neutralising antibody resulted in a significant reduction in *Tci*-L4-ES-mediated suppression of ConA-induced proliferation, reducing the suppressive capacity of *Tci*-L4-ES by between 25 and 64%. These results indicated that suppression of Con A-induced proliferation by *Tci*-L4-ES involves IL-10 signaling.

### L4 ES-mediated lymphocyte suppression declines following *T*. *circumcincta* challenge

**Figure 7:**
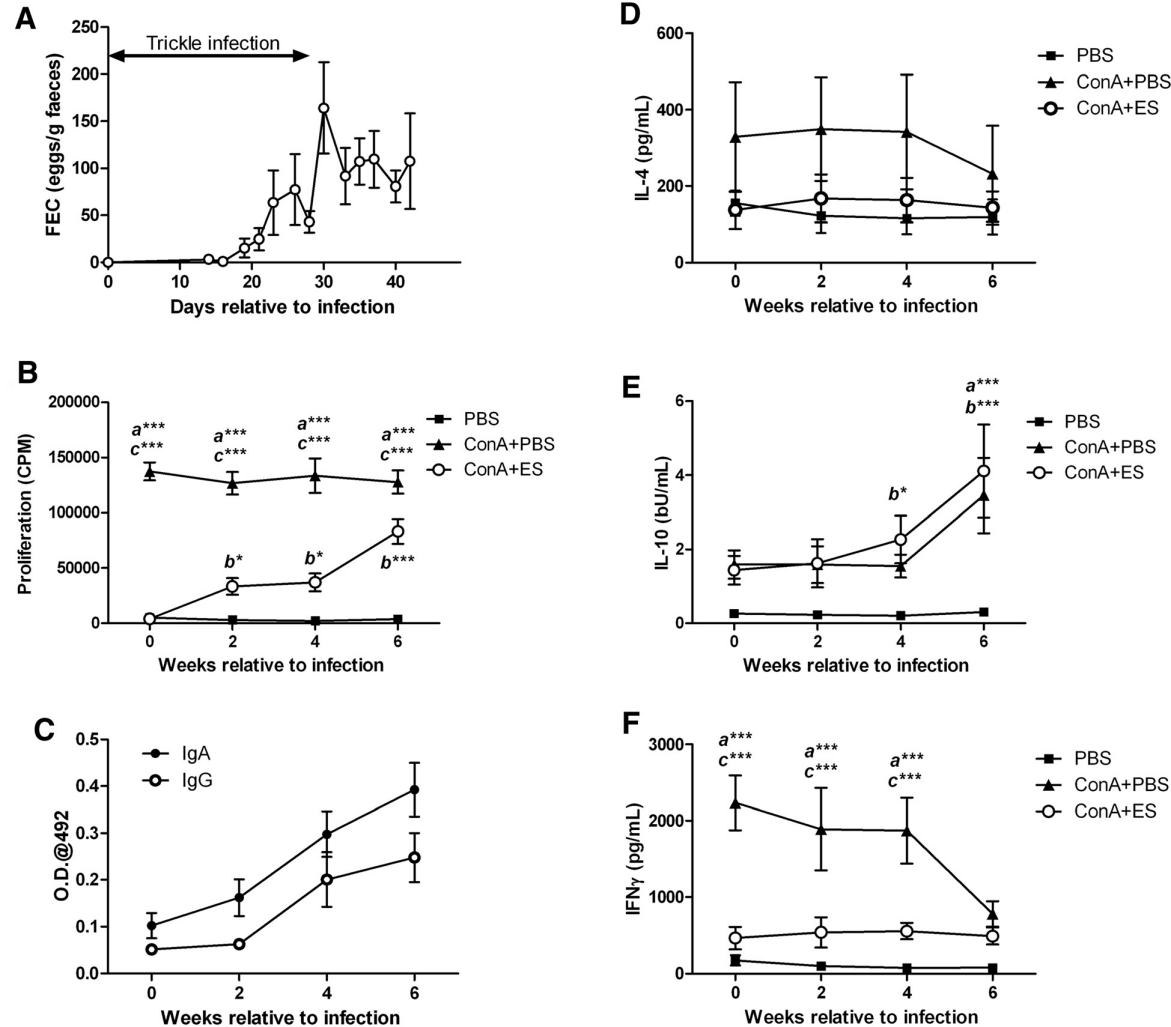
**The effects of *T. circumcincta *infection on *Tci*-L4-ES mediated lymphocyte suppression.** Seven helminth-naïve lambs were trickle infected with 2000 *T*. *circumcincta* L3 three times a week for four weeks. PBMC were harvested at 0, 2, 4 and 6 weeks from the start of infection and cultured for 72 h with PBS alone (PBS), 5 μg/mL Con A (Con A + PBS) or 5 μg/mL Con A + 15 μg/mL *Tci*-L4-ES (Con A + ES). Proliferation was assessed at 72 h by measurement of [^3^H] thymidine incorporation. Levels of cytokine present in the cell culture supernatants at 54 h were determined by ELISA. **(A)** Faecal egg count (FEC) data over the course of the experimental infection. **(B)** Proliferation of PBMC in response to PBS, Con A + PBS and Con A + ES during experimental *T*. *circumcincta* infection. **(C)** Levels of serum *Tci*-L4-ES-specific IgA and IgG during experimental *T*. *circumcincta* infection. **(D**-**F)** Concentrations of IL-4, IL-10 and IFN-γ in PBMC cultures supernatants. Data represents mean ± SEM. ^*a*^ significant difference between Con A + PBS vs. PBS-stimulated cultures; ^*b*^ significant difference between ConA + ES vs. PBS-stimulated cultures; ^*c*^ significant difference between Con A + PBS vs. Con A + ES stimulated cultures; *P < 0.05, ** *P* < 0.01, ***P < 0.001 (repeated-measures two-way ANOVA followed by the Bonferroni post hoc test for comparison of means).

The results above indicate that *Tci*-L4-ES is profoundly suppressive of Con A-induced activation and proliferation of PBMC that are derived from helminth-naïve lambs. To investigate the effects of *T*. *circumcincta* infection on *Tci*-L4-ES mediated suppression of PBMC in vitro, seven helminth-naïve lambs were infected repeatedly over a four-week period with *T*. *circumcincta* and the suppressive capacity of *Tci*-L4-ES on Con A-induced proliferation of PBMC harvested at 0, 2, 4 and 6 weeks from the start of infection (pi) determined. To confirm infection, FECs were recorded (Figure 7A). Proliferation of PBMC in response to Con A was consistent throughout the infection period at approximately 130 000 cpm (range = 127 000–137 000). In contrast, proliferation of PBMC in response to Con A + *Tci*-L4-ES increased from levels similar to un-stimulated cultures at week 0 (~ 4000 cpm) to around 83 000 cpm by week 6 pi (Figure 7B). Expressed as a percentage suppression of Con A-induced PBMC proliferation in matched control wells, this equated to a reduction from ~101% suppression at week 0 to ~36% suppression by week 6 pi. This reduction in the suppressive capacity of *Tci*-L4-ES coincided with the appearance of *Tci*-L4-ES-specific IgA and IgG antibodies in serum (Figure 7C).

We also determined levels of IL-4, IFN-γ and IL-10 in PBMC culture supernatants by ELISA. No significant differences in IL-4 secretion were observed between Con A, Con A + ES or un-stimulated cultures, although mean levels of IL-4 were always highest in Con A-stimulated cultures (Figure 7D). Similar IL-10 levels were observed in supernatants from Con A and Con A + ES stimulated cultures across all time-points and were greater than those observed in un-stimulated cultures. Across the sampling period, IL-10 levels remained similar at 0 and 2 week post infection (wpi), but increased in ConA + ES stimulated cultures at 4 and 6 wpi, and increased in Con A-stimulated cultures at 6 wpi (Figure 7E). Significantly higher levels of IFN-γ were present in Con A stimulated cultures compared to Con A + ES stimulated cultures at 0 to 4 wpi, with IFN-γ levels in Con A + ES-stimulated culture supernatants similar to those in un-stimulated cultures (Figure 7F). However, by 6 wpi, levels of IFN-γ in Con A-stimulated cultures had dropped and were no longer significantly different to those in ES or un-stimulated cultures.

**Figure 8:**
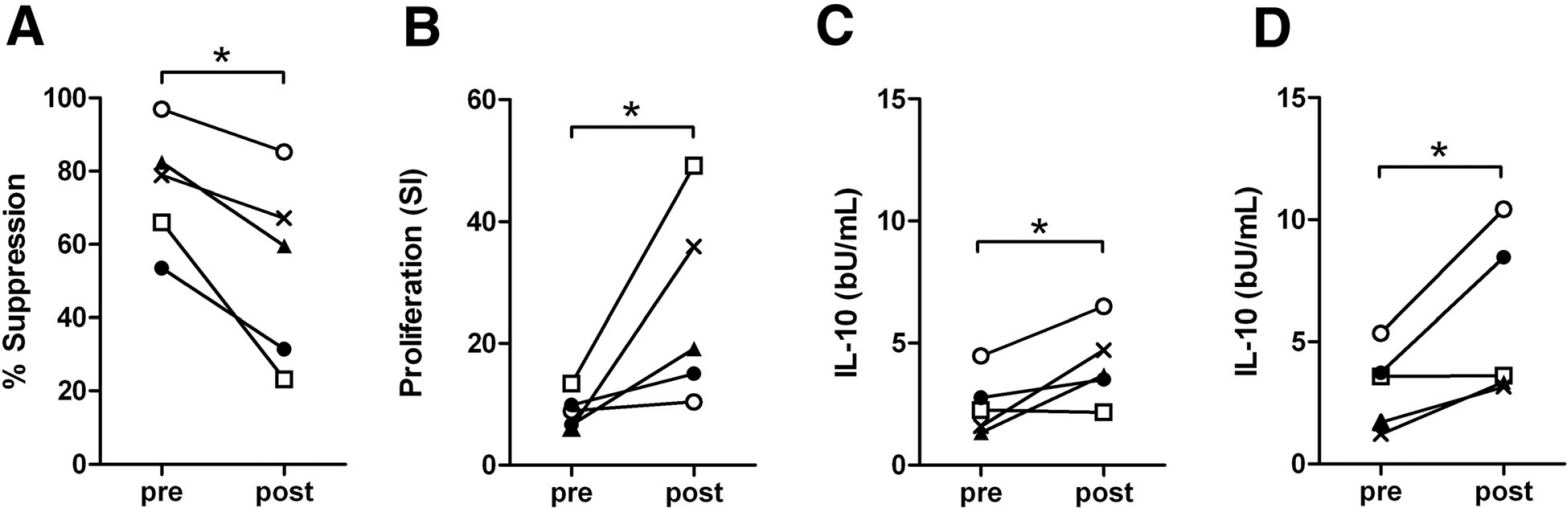
**The relationship between *Tci*-L4-ES mediated lymphocyte suppression and *Tci*-L4-ES antigen**-**specific lymphocyte proliferation.** Five helminth-naïve lambs were trickle infected with 2000 *T*. *circumcincta* L3 three times a week for six weeks. PBMC were harvested at 0 and 6 weeks from the start of infection and cultured for 72 h with PBS alone, 5 μg/mL Con A, 5 μg/mL Con A + 30 μg/mL *Tci*-L4-ES (Con A + ES) or 30 μg/mL heat-inactivated *Tci*-L4-ES (HiES). Proliferation was assessed at 72 h by measurement of [^3^H] thymidine incorporation. Levels of IL-10 present in the cell culture supernatants at 54 h were determined by ELISA. **(A)** Percentage suppression of Con A-induced proliferation of PBMC by ES. **(B)** Proliferation of PBMC in response to HiES expressed as a stimulation index (SI). **(C)** Concentration of IL-10 in culture supernatants of PBMC incubated with Con A + ES. **(D)** Concentration of IL-10 in culture supernatants of PBMC incubated with HiES. Pre = PBMC harvested before infection; Post = PBMC harvested after infection. Open squares represent data from the same animal. *P < 0.05 (students *t*-test).

One potential explanation for the reduced suppressive capacity of *Tci*-L4-ES on PBMC from *T*. *circumcincta* infected compared to helminth naïve lambs is that PBMC from infected lambs may contain increased numbers of *Tci*-L4-ES antigen-specific T cells which proliferate in response to antigens within *Tci*-L4-ES. To explore this further, five helminth-naïve lambs were repeatedly infected over a six-week period with *T*. *circumcincta* L3. PBMC were harvested immediately prior to infection and at 6 wpi and both the suppressive capacity of *Tci*-L4-ES on ConA-induced proliferation and proliferation in response to heat-inactivated *Tci*-L4-ES was determined. ES was heat-inactivated in this experiment to abrogate the suppressive effects of native ES while at the same time providing a source of ES antigens for T cells. The results are shown in Figure 8. As in the previous experiment, *Tci*-L4-ES was less able to suppress PBMC from *T*. *circumcincta* infected lambs compared to PBMC obtained prior to infection (Figure 8A, *P* < 0.05). This reduction in suppression of PBMC was associated with a significant increase in proliferation of PBMC in response to heat inactivated *Tci*-L4-ES antigen(s) (Figure 8B, *P* < 0.05). Furthermore, IL-10 levels in the culture supernatants were significantly increased in PBMC cultures from infected vs. non-infected lambs following stimulation with both Con A + *Tci*-L4-ES and heat-inactivated *Tci*-L4-ES (Figure 8C-D, both *P* < 0.05). Interestingly, PBMC from the lamb with the lowest decrease in ES-mediated suppression had the lowest increase in proliferation to heat-inactivated ES antigens, and also exhibited the lowest increase in IL-10 production following stimulation with ConA + ES or heat-inactivated ES.

## Discussion

Helminth infections are a major constraint to livestock production worldwide and represent a major challenge to continued food security. The parasitic nematodes that colonize the gastrointestinal tract of ruminants cause decreased appetite, reduced growth rate and persistent diarrhoea [36]. Virtually all ruminants grazing improved pastures are infected, either clinically or sub-clinically, with nematode members of the family Trichostrongylidae, of which *T*. *circumcincta* is the major genus found in sheep and goats in temperate areas. A feature of most gastrointestinal nematode infections is their chronicity, with poor immune responsiveness in young hosts [37] and/or parasite induced immune suppression [38] cited as major influences on the rate at which resistance to challenge develops. Because of the extensive nature of anthelmintic resistance, particularly in sheep nematodes [4], vaccines are being sought as alternatives for control. A major challenge to this research is the capability to design vaccines that are able to overcome the initial general lack of responsiveness to parasitic nematodes, particularly in young ruminants. This is concurrent with a time in life when producers are trying to maximize rates of growth. With this in mind, we have taken an approach to *T*. *circumcincta* vaccine development by investigating potential interactions between mucosal larvae and local effector responses. We have focused on larvae in the gastric gland (L4) because previous studies indicate that local humoral and cellular responses to these stages are critical in the initial acquisition of immunity in lambs [10,12,39,40]. Parasite ES products were targeted as they are the major interface between host and nematode. In studying the effect of *Tci*-L4-ES on lymphocytes, we have identified a novel mechanism by which this parasitic nematode appears to suppress lymphocyte activation and proliferation and hence may modulate the host adaptive immune response. This effect was particularly noticeable when we studied cells obtained from helminth naïve sheep and was reduced when we used cells derived from animals that were exposed to *T*. *circumcincta* infection, indicating that as animals develop immunity, the suppressive response associated with L4 ES components are reduced.

The induction of Foxp3^+^ regulatory T cells has been shown to be an important mechanism by which certain parasites down-regulate the host immune response to enhance survival [41-45]. In this study, we confirmed that numbers of Foxp3^+^ T cells are elevated in the abomasa of animals in the early stages of infection when L4 stage parasites are predominant. However, within the abomasal mucosa, the proportions of CD3^+^ T cells expressing Foxp3 did not change before and after infection suggesting that, rather than being specifically induced by the parasite, the observed Foxp3^+^ T cell response to *T*. *circumcincta* infection during these early stages of infection may reflect a more homeostatic regulatory mechanism within the abomasal cellular immune response to minimize immune-mediated abomasal pathology. This would be consistent with subsequent observations that *Tci*-L4-ES did not induce an increase in the proportion of activated ovine lymphocytes expressing Foxp3 in vitro. It should be noted that a relatively large single bolus infection (50 000 *T*. *circumcincta* L3) was used in this experiment which may not be truly reflective of parasite challenge in the field. Nevertheless, this infection protocol has been used widely in a number of previous studies (e.g. [13,46,47]) and is a useful model to explore the underlying mechanisms of immunity to *T*. *circumcincta* in helminth-naïve lambs as firstly, lower bolus infections of L3 have been shown to be poor at inducing abomasal immune responses in helminth-naïve lambs [48] and secondly, low dose repeated trickle infections, which more accurately reflect challenge conditions in the field, are complicated by the presence of multiple parasitic stages within the abomasum, which makes interpretation of immune responses in relation to specific parasitic stages more complicated.

The lack of obvious Foxp3 induction in ovine lymphocytes by *Tci*-L4-ES is in contrast to results obtained in a previous study in which stimulation of murine CD4^+^ T cells with a combination of α-CD3/ α-CD28 antibody, IL-2 and an identical concentration of *Tci*-L4-ES, resulted in ~20% increase in the proportion of cells expressing Foxp3 [15]. In the mouse study, induction of Foxp3 by *Tci*-L4-ES was attributed to TGF-β-like activity within the ES which is known to preferentially act on naïve CD4^+^ T cells to induce Foxp3 expression [15,33]. However, even culturing naïve CD4^+^ T cells (CD4^+^CD25^-^CD45RA^+^) with *Tci*-L4-ES failed to result in any increase in Foxp3 expression. The difference in the data between the mouse study and our own study may be due to a number of factors, including the use of purified CD4^+^ lymphocytes rather than PBMC cultures (as used here), different methods of polyclonal T cell stimulation (α-CD3/α-CD28 antibody and IL-2 compared to Con A), or inherent differences between murine and ovine lymphocytes such as differential sensitivities to TGF-β signaling or different requirements for induction of Foxp3 expression. However, the mouse study demonstrated that addition of *Heligmosomoides polygyrus* ES induced de novo Foxp3 expression in Con A-stimulated splenocyte cultures [15], a culture system similar to that employed here. Regardless of the cause of the differences in data between the two studies, the results here highlight the importance of utilizing cells derived from a parasite’s definitive host to accurately define the appropriate host-parasite interactions.

Rather than inducing Foxp3 expression, the major effect that *Tci*-L4-ES had on ovine T lymphocytes was profound suppression of mitogen-stimulated and antigen-specific lymphocyte proliferation which was heat-labile in nature. This was associated with a lack of up-regulation of IL2-Rα following mitogenic stimulation. As this receptor is expressed at an early stage in T cell activation [49], this indicates that *Tci*-L4-ES may interfere with early activation events. While the exact mechanism is still to be elucidated, suppression appeared to be associated with up-regulation of IL-10 transcription and was partially blocked by specific monoclonal antibody neutralisation of IL-10. The latter is an anti-inflammatory and immunomodulatory cytokine produced my several immune cell types (T cells, B cells and monocyte/macrophages) and is capable of suppressing T lymphocyte responses though a number of mechanisms. For example, IL-10 down-regulates expression of major histocompatibility complex class II proteins and co-stimulatory molecules, such as CD80 and CD86, on the surface of antigen presenting cells [50,51], thereby interfering with the generation and maintenance of T cell responses. IL-10 is also known to directly suppress CD4^+^ T cell proliferation by inhibiting IL-2 gene transcription [52]. The cellular source of IL-10 in our assays remains to be determined. We also observed a significant decrease in IL-4 transcription in Con A stimulated lymphocyte cultures when *Tci*-L4-ES was present. This may have biological relevance as protective immunity to *T*. *circumcincta* has been associated with Th2-type responses [13]. This effect was heat-stable indicating that different components within *Tci*-L4-ES are capable modulating IL-10 and IL-4 gene expression. Given the heat-stable nature of the ES-mediated down-regulation of IL-4, it is possible that contaminating LPS was responsible for the observation, as LPS is relatively heat-stable and known to polarize towards a Th-1 type response [53].

While this is the first observation of lymphocyte suppression by an ovine parasitic nematode, a number of related pathogens exert similar effects in other species. Both ES products and somatic extracts from *O*. *ostertagi* L4 have been shown to suppress Con A-induced proliferation of bovine lymphocytes [17]. In this case, suppression was similarly associated with reduced expression of IL2-Rα and up-regulation of IL-10 gene expression. *H*. *polygyrus* ES has been shown to suppress both antigen-specific antibody production and lymphocyte proliferation in vitro [54,55] and *Nippostryongylus brasiliensis* adult ES was also demonstrated to suppress mitogen-induced proliferation [56]. With regard to ovine helminths, ES material derived from the ovine trematode, *Fasciola hepatica*, has been shown to be capable of suppressing mitogen-induced proliferation of both ovine and human lymphocytes [57]. One important question which relates to all in vitro experiments with parasite ES is how relevant the concentrations of ES used in vitro are to those encountered in vivo. While it is difficult to determine the exact concentration of *Tci*-L4-ES encountered by ovine lymphocytes in vivo, which may be influenced by both the proximity of lymphocytes to the parasite and the quantity of ES generated in vivo, the concentrations of ES used in this study were identical to those used in the murine study of *Tci*-L4-ES [15], and were not high enough to affect cell viability, neither inducing apoptosis or necrosis in the PBMC cultures.

One salient result here was the observed reduction in the suppressive capacity of *Tci*-L4-ES when the parasite extract was used in combination with PBMC derived from sheep which had been trickle infected with *T*. *circumcincta* to mimic challenge conditions in the field. The observed reduction in suppression was associated with an increase in circulating *Tci*-L4-ES-specific antibodies, an increase in *Tci*-L4-ES-antigen specific T cell proliferation, and occurred at a time in the infection protocol where we would have expected immune-induced inhibition of L4 development [5]. The reduced “suppressive” effect of *Tci*-L4-ES on lymphocytes from infected sheep was thus likely associated with development of adaptive immunity. This indicates that despite obvious suppression of T cell activation by *Tci*-L4-ES in vitro, any potential suppressive effect that *Tci*-L4-ES exhibits in vivo was not sufficient to prevent the generation of *Tci*-L4-ES antigen specific T cell responses during *T*. *circumcincta* infection. Whether *Tci*-L4-ES has a modulating effect on the acquisition of immunity against *T*. *circumcincta*, for example by causing a delay in the development of parasite-specific adaptive immune responses, is as yet unclear.

During the first 4 weeks of experimental infection, cultures stimulated with Con A + ES released relatively more IL-10 and less IFN-γ, than cultures stimulated with Con A alone, consistent with the previous observation that *Tci*-L4-ES appears to induce increased relative gene expression of IL-10 in PBMC cultures. The lack of a significant increase in total IL-10 protein release from Con A + ES stimulated cultures compared to those stimulated with Con A alone may be explained by the observation that throughout the infection study, Con A + ES-stimulated cultures proliferated and therefore expanded to a lesser extent than those cultured with Con A without ES, and therefore produced relatively more IL-10 per cell than non-ES stimulated cultures. As Con A + ES stimulated cultures were observed to proliferate, there was an increase in IL-10, but not IFN-γ production, suggesting that proliferating ES-antigen specific lymphocytes exhibited a Th-2 or regulatory phenotype. This was consistent with the observation that PBMC from infected lambs released more IL-10 following stimulation with heat-inactivated ES antigen compared to PBMC obtained prior to infection, indicating that proliferating ES-antigen specific lymphocytes secreted IL-10. That PBMC cultures at later time-points during infection which were less suppressed by *Tci*-L4-ES released more IL-10 seems somewhat contradictory to our previous findings that IL-10 signaling appeared to be an important mechanism of suppression. However, it may be that the timing of IL-10 signaling, rather than the total levels of IL-10, is critical for suppression. It also suggests that additional mechanisms other than IL-10 signaling are involved in the suppressive effects of *Tci*-L4-ES.

While proliferation levels of PBMCs to Con A remained constant throughout the *T*. *circumcincta* infection, by 6 wpi there appeared to be a switch in cytokine production from IFN-γ to IL-10. This down-modulation of IFN-γ production towards a more IL-10-dominated response has been demonstrated in other nematode infections [58,59] and may have profound implications for individuals co-infected with pathogens for which IFN-γ/Th-1 type responses are associated with protective immunity. Such pathogens include a number of important endemic diseases of sheep including *Chlamydophila abortus*[60], *Toxoplasma gondii*[61] and *Mycobacterium avium subspecies paratuberculosis*[27] and it is possible that co-infection with *T*. *circumcincta* may have an effect on an individual’s ability to cope with these pathogens. Therefore, in addition to the production losses associated with the parasite itself, *T*. *circumcincta* infections may have far wider consequences for sheep flock health.

In conclusion, this is the first study to identify immunosuppressive activity in ES products derived from an ovine parasitic nematode. While caution must be taken in extrapolating *in vitro* data to the more complex situation in vivo and the exact significance of *Tci*-L4-ES mediated immune suppression in vivo has yet to be determined, the immunosuppressive effects of *Tci*-L4-ES may represent a mechanism by which *T*. *circumcincta* interferes with the generation of protective immune responses in vivo, facilitating survival of the parasite within the host. Consequently, immunosuppressive molecules within *Tci*-L4-ES may be useful vaccine targets, a concept that is supported by the demonstration that immunization of cattle and sheep with the immunosuppressive protein cathepsin L from *Fasciola hepatica* induces significant levels of protection against this trematode [62]. Furthermore, the modulation of lymphocyte cytokine responses during *T*. *circumcincta* infection may have wider consequences on the susceptibility of individuals to other infectious agents and further emphasizes the need for adequate control of this parasitic nematode.

## Competing interests

The authors declare that they have no competing interests.

## Authors’ contributions

TM designed the experiments, performed immunological and statistical analyses and drafted the manuscript. MR performed flow cytometry analyses. YB and LM carried out the parasitological analyses and assisted with animal experiments. DF carried out immunological analyses, ES preparation and assisted with animal experiments. JM and JKB performed immunohistochemical analyses. CL performed serological analyses. SW performed immunological analyses. AJN, JFH and JBM contributed to the design of the experiments and drafting of the manuscript. All authors read and approved the final manuscript.

## Supplementary Material

Additional file 1 **Effect of LPS removal on suppression of mitogen-induced lymphocyte proliferation by *Tci*-L4-ES.** To determine whether the effects of *Tci*-L4-ES on mitogen-induced proliferation were due to the presence of contaminating LPS, PBMC from a helminth-naïve lamb were cultured with 5 μg/mL Con A in the presence or absence of 30 μg/mL *Tci*-L4-ES without LPS removal (ConA+ES) or 30 μg/mL *Tci*-L4-ES in which LPS had been removed (ConA+LPS-free ES). Proliferation was assessed by incorporation of [3H] thymidine at 72 h culture and expressed as counts per minute (cpm). Addition of both ES and LPS-free ES resulted in a significant reduction in proliferation compared to PBMC cultures stimulated with Con A alone. However, no significant difference in proliferation was observed between ConA+ES and ConA+LPS-free ES stimulated cultures. Data represents mean ± SEM from three replicate cultures. ***P < 0.001 (one way ANOVA followed by the Tukey *post hoc* test for pairwise comparison of means). Click here for file

Additional file 2 **Foxp3 expression in naïve CD4**^**+**^** T cells following stimulation with Con A and *Tci*-L4-ES.** 5 × 10^4^ FACS sorted naïve CD4^+^CD25^-^CD45RA^+^ T cells from a helminth-naïve lamb were cultured with 1 × 10^5^ irradiated autologous antigen-presenting cells with PBS alone (PBS), 5 μg/mL Con A alone (ConA+PBS) or 5 μg/mL Con A + 30 μg/mL *Tci*-L4-ES (ConA+ES). After 72 h cells were labeled with anti-CD4-FITC and anti-Foxp3-PE-Cy7 antibodies and analysed by flow cytometry. Flow cytometry was performed in parallel on PBMC from the same lamb to validate the flow cytometry technique. (A) Representative plots of Foxp3 expression by CD4^+^ T cells following stimulation with ConA ± *Tci*-L4-ES. (B) Percentages of CD4^+^ cells expressing Foxp3 from triplicate experiments. No significant difference in% Foxp3 expression was seen (one way ANOVA). Data represents the mean ± SEM. (C) Control flow cytometry labeling of PBMC for CD4 and Foxp3. Click here for file
