# Supplementary figures and images for: Suppression of ovine lymphocyte activation by Teladorsagia circumcincta larval excretory-secretory products
Source: Vet Res. 2013 Aug 21;44(1):70. doi: 10.1186/1297-9716-44-70 (PMC3848371; doi:10.1186/1297-9716-44-70)

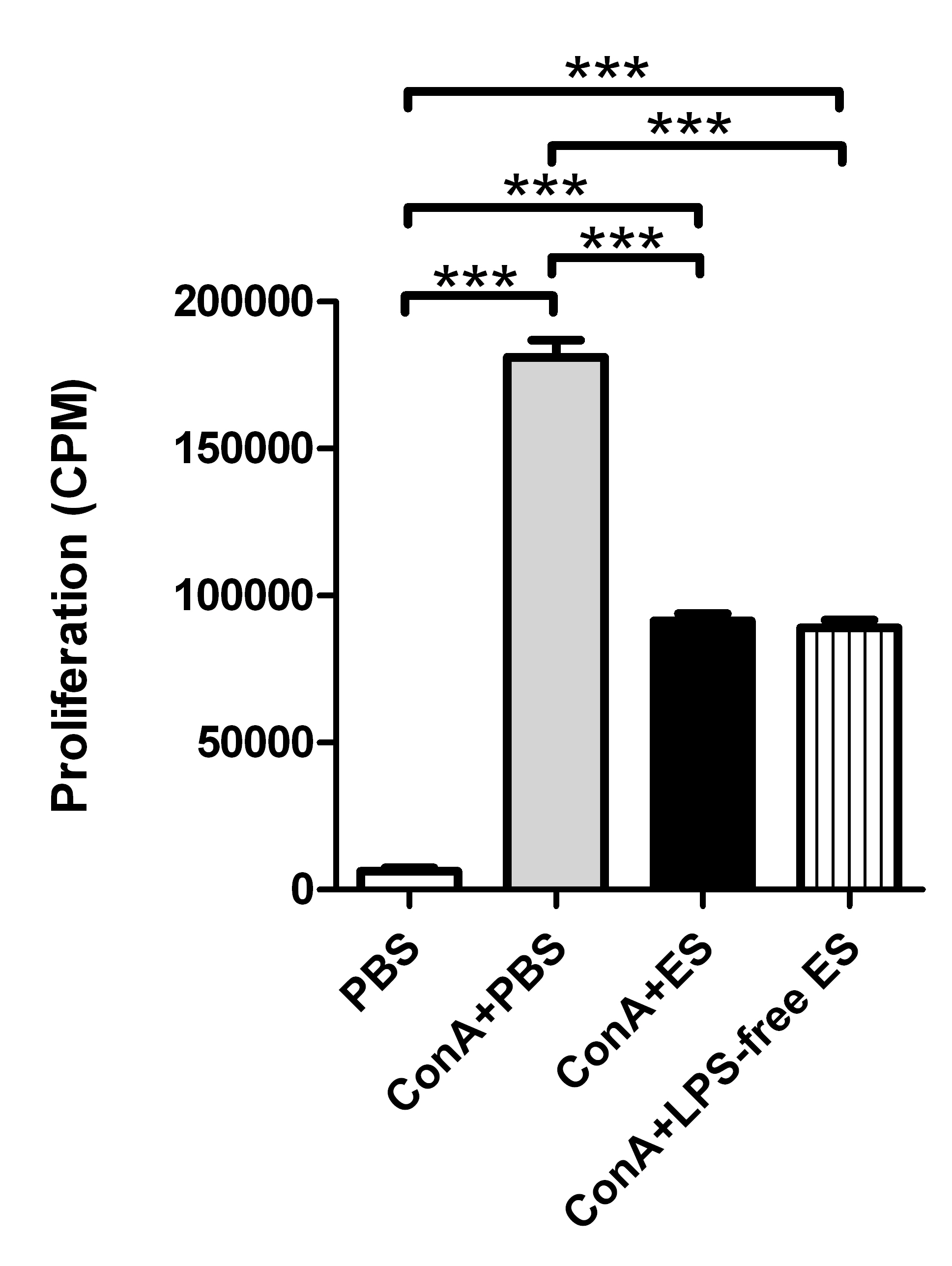

Supplement: Additional file 1 — Effect of LPS removal on suppression of mitogen-induced lymphocyte proliferation by Tci-L4-ES. To determine whether the effects of Tci-L4-ES on mitogen-induced proliferation were due to the presence of contaminating LPS, PBMC from a helminth-naïve lamb were cultured with 5 μg/mL Con A in the presence or absence of 30 μg/mL Tci-L4-ES without LPS removal (ConA+ES) or 30 μg/mL Tci-L4-ES in which LPS had been removed (ConA+LPS-free ES). Proliferation was assessed by incorporation of [3H] thymidine at 72 h culture and expressed as counts per minute (cpm). Addition of both ES and LPS-free ES resulted in a significant reduction in proliferation compared to PBMC cultures stimulated with Con A alone. However, no significant difference in proliferation was observed between ConA+ES and ConA+LPS-free ES stimulated cultures. Data represents mean ± SEM from three replicate cultures. ***P < 0.001 (one way ANOVA followed by the Tukey post hoc test for pairwise comparison of means). [file 1297-9716-44-70-S1.jpeg]

## Slide 1
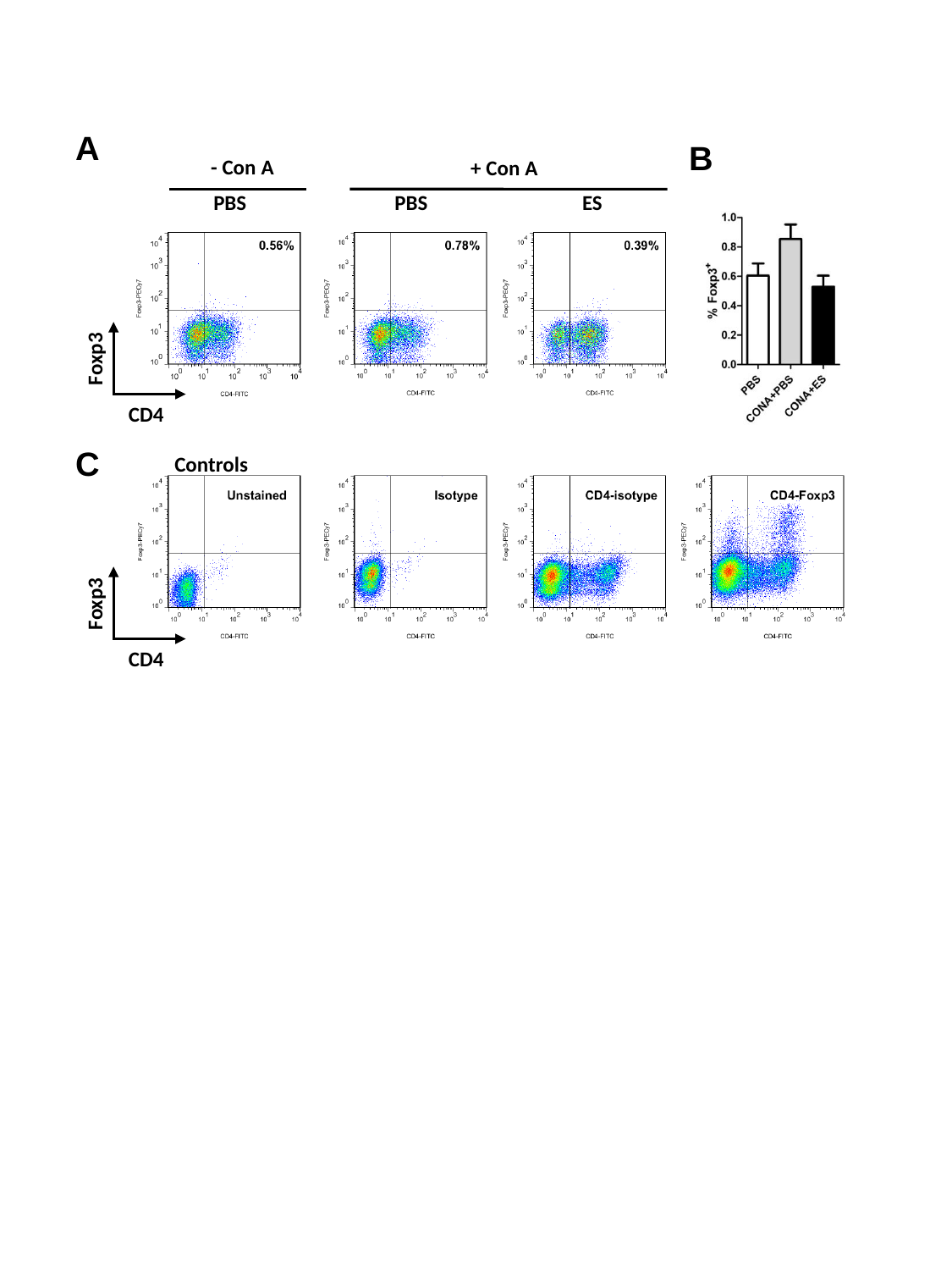

A
B
- Con A
+ Con A
PBS
PBS
ES
CD4+CD25-CD45RA+
Foxp3
CD4
C
Controls
Foxp3
CD4

Supplement: Additional file 2 — Foxp3 expression in naïve CD4+ T cells following stimulation with Con A and Tci-L4-ES. 5 × 10^4^ FACS sorted naïve CD4+CD25-CD45RA+ T cells from a helminth-naïve lamb were cultured with 1 × 10^5^ irradiated autologous antigen-presenting cells with PBS alone (PBS), 5 μg/mL Con A alone (ConA+PBS) or 5 μg/mL Con A + 30 μg/mL Tci-L4-ES (ConA+ES). After 72 h cells were labeled with anti-CD4-FITC and anti-Foxp3-PE-Cy7 antibodies and analysed by flow cytometry. Flow cytometry was performed in parallel on PBMC from the same lamb to validate the flow cytometry technique. (A) Representative plots of Foxp3 expression by CD4+ T cells following stimulation with ConA ± Tci-L4-ES. (B) Percentages of CD4+ cells expressing Foxp3 from triplicate experiments. No significant difference in% Foxp3 expression was seen (one way ANOVA). Data represents the mean ± SEM. (C) Control flow cytometry labeling of PBMC for CD4 and Foxp3. [file 1297-9716-44-70-S2.pptx]
